# Mental Health Care Navigation Tools in Australia: Infoveillance Study

**DOI:** 10.2196/60079

**Published:** 2024-11-22

**Authors:** Cindy E Woods, Mary-Anne Furst, Manoj Dissanayake, Jane Koerner, Carlota de Miquel, Sue Lukersmith, Sebastian Rosenberg, Luis Salvador-Carulla

**Affiliations:** 1 Faculty of Health, Health Research Institute, University of Canberra, 11 Kirinari Street, Bruce, 2617, Australia, 61 0449181321; 2 Research, Innovation and Teaching Unit, Parc Sanitari Sant Joan de Déu, Sant Boi de Llobregat, Spain; 3 Medicine and Translational Research, University of Barcelona, Barcelona, Spain; 4 Centro de Investigación Biomédica en Red de Salud Mental, Madrid, Spain

**Keywords:** digital health, infoveillance, mental health, mental health care, navigation tools, Australia, fragmentation, digital mental healthcare, web-based digital resources, diagnostic screening, accessibility, user friendly

## Abstract

**Background:**

In response to the well-documented fragmentation within its mental health system, Australia has witnessed recently rapid expansion in the availability of digital mental health care navigation tools. These tools focus on assisting consumers to identify and access appropriate mental health care services, the proliferation of such varied web-based resources risks perpetuating further fragmentation and confusion for consumers. There is a pressing need to systematically assess the characteristics, comprehensiveness, and validity of these navigation tools, especially as demand for digital resources continues to escalate.

**Objective:**

This study aims to identify and describe the current landscape of Australian digital mental health care navigation tools, with a focus on assessing their comprehensiveness, identifying potential gaps, and the extent to which they meet the needs of various stakeholders.

**Methods:**

A comprehensive infoveillance approach was used to identify Australian digital mental health care navigation tools. This process involved a systematic web-based search complemented by consultations with subject matter experts. Identified navigation tools were independently screened by 2 authors, while data extraction was conducted by 3 authors. Extracted data were mapped to key domains and subdomains relevant to navigation tools.

**Results:**

From just a handful in 2020, by February 2024 this study identified 102 mental health care navigation tools across Australia. Primary Health Networks (n=37) and state or territory governments (n=21) were the predominant developers of these tools. While the majority of navigation tools were primarily designed for consumer use, many also included resources for health professionals and caregivers. Notably, no navigation tools were specifically designed for mental health care planners. Nearly all tools (except one) featured directories of mental health care services, although their functionalities varied: 27% (n=27) provided referral information, 20% (n=21) offered geolocated service maps, 12% (n=12) included diagnostic screening capabilities, and 7% (n=7) delineated care pathways.

**Conclusions:**

The variability of navigation tools designed to facilitate consumer access to mental health services could paradoxically contribute to further confusion. Despite the significant expansion of digital navigation tools in recent years, substantial gaps and challenges remain. These include inconsistencies in tool formats, resulting in variable information quality and validity; a lack of regularly updated service information, including wait times and availability for new clients; insufficient details on program exclusion criteria; and limited accessibility and user-friendliness. Moreover, the inclusion of self-assessment screening tools is infrequent, further limiting the utility of these resources. To address these limitations, we propose the development of a national directory of mental health navigation tools as a centralized resource, alongside a system to guide users toward the most appropriate tool for their individual needs. Addressing these issues will enhance consumer confidence and contribute to the overall accessibility, reliability, and utility of digital navigation tools in Australia’s mental health system.

## Introduction

### Background

In Australia, as in many developed countries, the mental health care system is characterized by increasing fragmentation [1]. Responsibilities for mental health policy and services are split between our federal government, which funds primary mental health care services, and our eight state and territory governments which are responsible for hospital-based emergency, acute, and outpatient mental health care. Myriad state and national mental health plans and strategies have not fundamentally shifted a situation in which mental health care receives about 7% of the total national health spending [2] (a figure unchanged since 1992) while accounting for around 15% of the total burden of disease [3].

This mix of responsibilities has seen a withering of community mental health services over time, creating a “missing middle” in Australia’s mental health service landscape [4].

Fragmentation also arises through disconnection between public and private mental health sectors, very significant mental health workforce shortages, and a heavy reliance on the medical model, which favours clinical and hospital-based care over community and psychosocial mental health services. Psychosocial services have always been a peripheral element of Australia’s mental health service mix [5].

Australia’s recent implementation of its National Disability Insurance Scheme has also created a new funding and policy seam separating different mental health clients and systems.

At the same time, and in response to increasing recognition of the need for more services to respond to mental illness in Australia, funding has gone into a plethora of new programs. Often these programs are time-limited, operating in isolation from one another. Repeated inquiries and reports have found these issues and others combine to make mental health system integration very difficult in Australia [6,7].

New navigation tools offer some prospect of helping surmount this fragmentation. In doing so, they must fit into, or change the current model of primary mental health care provided in Australia, which sees the general practitioner (GP) operate as a gatekeeper to publicly subsidized, specialist mental health care, typically provided by either a psychologist or psychiatrist under Medicare.

Australians cannot directly book Medicare‐subsidized sessions with psychologists or other mental health care professionals without a referral from a primary care provider [8]. There is no suggestion (yet) that navigation tools should supplant the GP gatekeeper role in Australia. However, they can still play a valuable role, helping consumers and caregivers to find community mental health services or to advocate for a referral to specialist care. Additionally, health professionals, including primary care providers and mental health planners, can benefit from using these tools to support and streamline care pathways.

Navigation tools help health care service planners, health professionals, and health care consumers locate available services [5,9] and connect individuals and their families with the most suitable option for mental health treatment and support services. In the last few years, the number of Australian digital mental health care navigation tools has significantly increased from a handful in 2020 to currently over 100. This abundance of web-based and digital resources can be overwhelming and confusing to navigate due to the volume of information, variable levels of digital literacy, and uncertainty about which services are appropriate [10]. With growing demand for digital resources in mental health, it is crucial to gain a better understanding of current digital navigation tools.

In the context of this study, mental health care navigation tools are defined as digital or web-based resources designed to assist health professionals, consumers, and caregivers in locating and accessing mental health services, referral information, resources, and support. These tools can exist as stand-alone web-based systems or modules integrated with other web-based tools for assessment, diagnosis, and facilitation of appropriate treatment. Navigation tools commonly feature directories of mental health services and offer interactive, integrated, and user-friendly functions for locating resources and support. They may also provide estimated appointment waiting times, web-based booking services, telehealth platforms, relevant information pertaining to rules about clients or restrictions, and digital consultations.

Navigation tools are not “navigators” of the care system. There is a widespread interchangeability and confusion, even in the scientific literature, of navigation as a function or activity, the tools used for conducting navigation activities, and the specific occupation of “navigators” who perform care coordination activities using publicly or organizationally available tools. Studies seldom provide clear definitions of their operational use of the term navigation and often conflate navigation activities with the role of “navigators” [11,12].

In Australia, as with other developed nations, there are various ways to access the mental health care system. However, access often relies on individuals being aware of these options. Having all service choices available in one location can help overcome barriers related to lack of knowledge of available services and save consumers time in trying to determine what is accessible in their area [13]. One of the benefits of digital mental health care navigation tools is that they provide aggregated information about available resources in a centralized location to help navigate the complex mental health system. The tools are often stratified by geographic location, making it easier for health professionals and consumers to locate appropriate services [14].

Navigation tools can also be used by health care and support workers, care coordinators, and care navigators to provide recommendations about mental health care services and guide referrals. Digital mental health care navigation tools can offer insights into specific demographic needs by leveraging metric analytics to understand usage patterns and inform policy and program planning, ensuring that services are tailored to meet the specific needs of different populations [15].

However, access to and use of digital mental health care navigation tools depends on end user awareness and other factors such as their level of digital literacy. The use of digital supports may create additional challenges for those in most need of care due to issues such as limited technology access, unreliable internet, associated costs, and lack of digital and health literacy [16].

Currently, there is an absence of standardized evaluation criteria for conducting reviews and assessing the quality and accuracy of digital navigation tools. The National Safety and Quality Digital Mental Health Standards (NSQDMHS) are a set of guidelines developed in Australia to ensure high-quality mental health care across the country. Their purpose is to establish a benchmark for the quality and safety of mental health services and to guide service providers in delivering consistent and effective mental health care. The standards are organized into several core areas that outline the expectations for mental health services. These core areas are consumer rights and safety, access and engagement, treatment and support, service environment, continuity of care, and workforce. The NSQDMHS [17] provides voluntary standards for digital mental health services. These standards do not explicitly address mental health navigation tools, nor do they encompass principles of quality and accuracy for information within digital navigation tools. The standards suggest that service providers use quality improvement systems to identify quality measures, but do not offer guidance on definitions of quality or accuracy.

Infoveillance, a combination of information and surveillance, refers to the systematic collection, analysis, and monitoring of information from digital sources [18,19]. Infoveillance can also be used to take a snapshot of health system change by analyzing data from various web-based sources [20], allowing for repeated assessments at different time points. Infoveillance allows researchers and others to gather insights from digital data to gain a better understanding of various phenomena [21].

Analyzing existing mental health service navigation tools using the infoveillance method can help to identify availability gaps and access barriers to inform the development of more inclusive and accessible tools. Additionally, it can offer valuable insights for policymakers to comprehend the mental health landscape and address support gaps effectively [18].

### Aim

The aim of this study was to identify and outline the primary features of existing Australian digital mental health care navigation tools while pinpointing gaps in availability and barriers to access.

## Methods

### Search Criteria

The research team conducted an infoveillance study to identify Australian mental health care navigation tools. A comprehensive web search was conducted by 2 members of the research team (CEW and MAF). Primary Health Networks (PHNs), the 31 regional districts of primary health care in Australia, were used as the reference catchment areas. We conducted a literature search, reached out to key experts to identify available tools, and performed a comprehensive Google web search to locate mental health navigation tools at both the local and national levels. This involved reviewing the official web pages of the PHNs as well as any other available sources of regional-level information.

The web search was executed using Google, incorporating the name of the PHN along with the following search terms and Boolean operators: AND directory OR navigation. The search results initiated a snowball effect, with some PHNs providing links on their website to state-based or national navigation tools. Concurrently, a systematic scoping review search was conducted alongside the infoveillance search to identify literature pertaining to navigation tools. The scoping review was not part of our study, but it was instrumental in determining if any navigation tools were overlooked in the infoveillance search.

### Inclusion and Exclusion Criteria

Inclusion criteria were specific mental health navigation tools, or general health navigation tools that contain mental health and related services such as those for people experiencing homelessness, financial crisis, substance use, and gambling. Exclusion criteria were tools that do not have any form of service directory, screening, pathways, or referral information on their website**,** and navigation services that are staffed by care navigators who screen and connect an individual to an appropriate support service.

### Development of a Data Extraction Tool

The research team developed a framework with relevant domains and subdomains of navigation tool features and characteristics a priori, drawing on an author’s digital mental health expertise (LSC). This predetermined set of domains and subdomains, along with the definition of navigation tools, was assessed by a multidisciplinary expert panel (n=19), which met twice via video-conferencing and provided feedback and external validation. Experts were invited to participate in the expert panel via email. Invitations were sent to networks known to the authors and also to targeted experts in mental health services planning and PHNs, clinically experienced mental health practitioners (psychologists and psychiatrists), mental health community-based organization representatives, and an advocate with lived experience.

The expert panel consisted of representatives from peak mental health bodies, government health or mental health departments, consumer and lived experience advocacy, the PHN, and GPs, psychologists, psychiatrists, and community mental health care providers. Based on the feedback from the panel, additional domains and subdomains were added to the framework. The domain of service information (waiting time and accessibility), and the subdomain of quality—information update frequency—were added to the framework. The expert panel identified a set of key components of a navigation tool relevant to evaluate its usability. These components related to technical aspects (interactivity) and a glossary of terms.

An Excel (Microsoft Corp) data extraction spreadsheet was used as the data extraction tool, with each subdomain listed as a separate column heading. The validated domains and subdomains include the following (Table 1):

**Table 1.** Navigation tool domains and subdomains used for the data extraction tool.

Navigation tool domains	Subdomains
Type	Target audience—health care service planners, health professionals, consumers, or caregivers Scope—general (includes mental health or other community services) or specific to a population group or health condition
Technical design aspects	Modules—directory, geolocation, screening, pathways, or referral Interactivity—interactive or static
Service information	Waiting time Accessibility
Quality	Glossary Information update frequency

### Data Extraction

Each navigation tool was independently screened against the inclusion or exclusion criteria by two authors (CEW and MAF). Three authors (CEW, MAF, and MD) made an independent evaluation of the dataset and individually extracted data regarding the subdomains, features, and links (or a representative sample if there were many) of each navigation tool. Any additional relevant features or characteristics identified during the data extraction process, such as quick exit buttons or symptom checkers, were subsequently incorporated into the data extraction form.

### Ethical Considerations

This study was reviewed and approved by Australian Capital Territory Health Human Research Ethics Committee (2023.ETH.00112). Participants were provided with an information sheet and signed a consent form before taking part in the expert panels. No compensation was provided to participants. Expert panel data were deidentified.

## Results

### Overview

The Google search identified 197 navigation tools in the 31 Australian PHNs. After removing duplicate national navigation tools that provide customized local navigation services to individual PHNs such as Head to Health, HealthPathways, and My Community Directory, 102 unique navigation tools remained. Table 2 shows the number of tools in each jurisdiction. The states with the highest population, namely New South Wales (PHN n=10) and Victoria (PHN n=6), have the largest quantity of navigation tools.

**Table 2.** Number of identified Australian national and state or territory navigation tools.

Location	Number of Primary Health Networks	Number of navigation tools
National	31	20
NSW^a^	10	26
VIC^b^	6	19
QLD^c^	7	11
ACT^d^	1	7
WA^e^	3	6
SA^f^	2	5
NT^g^	1	4
TAS^h^	1	4
Total	—^i^	102

a NSW: New South Wales.

b VIC: Victoria.

c QLD: Queensland.

d ACT: Australian Capital Territory.

e WA: Western Australia.

f SA: South Australia.

g NT: Northern Territory.

h TAS: Tasmania.

i Not applicable.

### Developers of Navigation Tools

Seven navigation tools were developed by the Australian Government, 3 were developed by a combination of Australian and state or territory governments, 21 were developed by state or territory government, 7 by local government (councils), and 1 by a New Zealand district health board. Three navigation tools were developed by a local health district while 37 were developed by one or more PHNs. Other developers include charities (n=7), not-for-profit or nongovernment organizations (n=5), community organizations (n=3), and a social enterprise (n=1). Private sector organizations developed 4 navigation tools and 2 were developed by a university.

### Costs

The majority of navigation tools are free to use although some do require registration. Two navigation tools provide a paid subscription service for health services or health care professionals that includes web-based service listings, web-based appointment bookings, digital advertising, and social media marketing, depending on the chosen subscription level. The use of one New Zealand–based navigation tool is funded by PHNs, and individual health care professionals or services are provided with access information. One navigation tool developed by large public corporations is free for consumers, caregivers, and health professionals to use, but fees are charged to provide anonymous search or usage data.

### Target Audience

#### Health Care Service Planners

There is considerable overlap with the target audiences of the tools (Table 3). For example, all of the navigation tools that contain information for planners (n=18), also provide information, resources, referrals, data or tools for health professionals, and a service directory for consumers (n=13) or caregivers (n=6). There were no navigation tools specifically for planners.

**Table 3.** Australian navigation tools for mental health care: target audiences and included modules.

Navigation tools		Target audiences or included modules, n
**Navigation tool target audiences**		
	Planners	18
	Health professionals	71
	Consumers	76
	Caregivers	50
**Navigation tool specific target audiences**		
	Planner specific	0
	Health professionals specific	11
	Consumer specific	15
	Caregiver specific	9
**Navigation tool modules**		
	Composite	45
	Directory	101
	Geolocation	21
	Pathways	7
	Referral	27
	Diagnostic screening tool	12

#### Health Care Professionals

The availability of information and resources for health care professionals varies significantly between the navigation tools. Of the 71 navigation tools with content for health professionals, eleven are tailored specifically to this audience, with one also providing data for planners. Some navigation tools provide the option for health care professionals to list or update their service (n=13), provide a widget or application programming interface (an interface which allows 2 computer software programs to communicate and share data or functionality) to use on their health service website, or provide information, resources, referral information, training options, data, or tools (n=44).

#### Health Consumers

##### Overview

The majority of navigation tools (n=76) are oriented toward mental health care service consumers, providing an extensive array of features including service listings with contact information, geolocation features, digital options (telehealth, ie, telephone and web-based consultations with health care professionals), screening tools, symptom checkers, risk assessments, accessibility options, and filtering features to identify the most appropriate services. There are a limited number of consumer-specific navigation tools (n=15) that do not provide any information for health care professionals or caregivers.

##### Caregivers

Nine navigation tools are specifically tailored to cater to the needs of caregivers, including formal and informal caregivers, parents, partners, and friends. The remaining navigation tools (n=41) either feature a section for caregivers or list some caregiver services. While these tools may primarily target consumers rather than caregivers specifically, it is assumed that caregivers will use the navigation tools to locate services for the individuals in their care.

##### Scope

The majority of navigation tools (n=70) are specific to a particular population or health condition (with some overlap) such as adult mental health (n=37), youth and adolescent mental health (n=3), caregivers (n=9), First Nations (n=3), health care professionals (n=11), dementia (n=3), alcohol and other drugs (AOD; n=13), disability (n=2), neurodevelopmental or behavioral disorders (n=1), or community services relevant to mental health such as homelessness, trauma, or family violence (n=7). The general navigation tools (n=32) were included if there was a section for mental health care services or a function to filter or search for mental health care or caregiver services.

### Navigation Tool Modules

#### Overview

Table 3 provides a summary of the following modules of mental health care navigation tools.

#### Composite

Composite navigation tools include more than one module. Around 44% (45/101) of navigation tools comprise various elements, including a directory, a map with geolocated services, care pathways, referral information, or a screening tool.

#### Service Directory

A service directory serves as a centralized point through which end users can access and locate various services or resources. In the past, directories typically lacked interactivity, such as search or filter functions. However, with the rapid expansion of digital navigation tools, directories are being enhanced with additional features. The large majority (n=101) of navigation tools include a service directory. Some are limited or basic (eg, name and contact details of the services) while others are comprehensive (eg, including accessibility information, public transport directions, or maps). As noted above, some navigation tools are general, including a broad range of community services, and some specific to a population group or health condition. The navigation tool that did not include a directory comprised the Initial Assessment and Referral Decision Support Tool (IAR-DST).

#### Geolocation

Just over 20% (21/101) of navigation tools include a map with geolocated services, thereby improving accessibility for individuals to easily find and access mental health care services in their vicinity or in close proximity to them. Other navigation tools (n=9) provide a map to locate individual services but do not provide a comprehensive map showing the location of all services within an area. None of the navigation tools incorporate a map directly on the landing page, and access to a map of services and locations necessitates between 1 and 6 mouse clicks.

#### Care Pathways

Care pathways, information to support assessment, management, and referral of consumers within the local health system, were included in 7% (n=7) of navigation tools. The majority (4/7) of these navigation tools are tailored specifically for health care professionals.

#### Referral

Twenty-seven navigation tools provide referral information on the process and criteria for accessing mental health care services, with significant variation observed between them. This variation ranges from information about whether a referral is needed and who can provide one (n=15), and outlining referral criteria (n=3), through to web-based referral forms and intake contacts (n=7). Some navigation tools use an IAR-DST level of care to guide referral decisions to appropriate care services. This tool supports the stepped care approach to service delivery, helping match individuals to the most suitable services for their mental health care needs.

#### Diagnostic Screening

Twelve navigation tools integrate a screening process, with some using a standardized screening tool, while others screen services based on the IAR level of care or incorporate screening into the referral process or form. The mental health screening tools include the Patient Health Questionnaire (measuring depression), the Generalized Anxiety Disorder Assessment (measuring anxiety), the Kessler Psychological Distress Scale (measuring distress), the Depression, Anxiety and Stress Scale (measuring depression, anxiety, and stress), and suicide and self-harm questions (n=1). The suicide and self-harm screening tool is administered by a health professional and cannot be accessed by consumers. The IAR-DST Guidance suggests using standardized assessment tools to measure symptom severity and distress such as the Kessler Psychological Distress Scale with additional questions measuring the impact of distress on daily living, Kessler Psychological Distress Scale for First Nation peoples, Patient Health Questionnaire, Generalized Anxiety Disorder Assessment, Edinburgh Postnatal Depression Scale, and the Work and Social Adjustment Scale to measure functional impairment pertaining to work and social functioning.

AOD screening tools include the Alcohol, Smoking and Substance Involvement Screening Test, the Alcohol Use Disorders Identification Test, Cannabis Use Disorders Identification Test, and substance use history and frequency questions.

The dementia screening tools include the Standardized Mini-Mental State Examination, dementia assessment tools, pain assessment tool, and caregiver burden scale.

All of the navigation tools with screening tools are specific rather than general. Two are specific to health care professionals (Health Pathways and the IAR-DST), 9 are specific to mental health or AOD, and 1 is dementia specific. Six navigation tools incorporate a self-assessment screening tool, and the remainder (n=6) are intended to be administered by health care professionals.

#### Other Characteristics: Estimated Wait Times and Accepting New Referrals

Forty-four percent (43/101) of navigation tools offer details regarding a service’s availability for new clients or estimates of the wait time for accessing the service. Among these, 22 navigation tools provide information about accepting new referrals and waiting time, 16 provide advice on accepting new or self-referrals but no information about waiting times, and 2 navigation tools advise estimated wait time but no information about accepting new referrals. Two of the navigation tools are specifically for health care professionals. One navigation tool provides a monthly subscription plan for service providers and integrates into the patient management system of a practice. Consumers can download the app or use the website to search for a service, choose a health professional by viewing their profile, see all available appointment times on different dates, and book an appointment on the web.

#### Access to Care

Fourteen navigation tools provide accessibility information such as wheelchair access, accessible or free parking, accessible toilet facilities, baby change facilities, pram friendly, and accessible telephone. The majority of these (n=9) are national navigation tools, and 5 of the 14 are mental health specific while the remainder are general health navigation tools.

Thirty-one navigation tools provide language options such as language translation, enlarged font, and audio screen reader. Out of these, 19% (19/101) are specific to a particular population or health condition such as mental health, disability, caregivers, dementia, aging, and AOD.

Four navigation tools have a chatbot or similar feature. However, one chatbot link was nonfunctional, another was linked to a council website for reporting storm damage, and one labeled “ask Cam, the carer navigator” functioned as a search tool. The only fully functional chatbot was part of an AOD service which also has a “text the effects” service, allowing users to text the name (or slang name) of any drug and be sent information about its effects. Additionally, five navigation tools offer a live chat facility or similar service. Of these, 3 connect users with AOD counselors while two facilitate chats with mental health professionals.

#### Accuracy

Out of the navigation tools that are interactive and provide links to service websites (n=77), 13 (17%) had one or more links that were broken. Thirty (30%) navigation tools did not provide any data about how often their information is updated. Of the navigation tools that communicate how often service information is updated (n=72), the frequency varied from last updated before 2020 (n=4), last updated in 2020‐2021 (n=3), updated in 2022 (n=8), updated in 2023 (n=16), and updated in 2024 (n=42). The National Health Service Directory has replaced manual data entry with data sharing agreements which enables them to obtain current information from established databases and authenticate directory details using Medicare and the Australian Health Practitioner Regulation Agency data. Ask Izzy (Infoxchange) uses a team of over 15 database updaters to ensure the ongoing accuracy and expansion of service listings. HealthPathways provides a “daily updates” web page which lists all reviews and updates of information for health care professionals, which occur weekly if not more frequently.

#### Interactivity

##### Search Functions

The majority of directories are interactive (n=80), and allow users to filter, screen, and search the digital information to identify and locate the services they need, while 22 are static lists of services with contact information. Some static directories provide links to service websites or links to other navigation tools which are interactive.

##### Feedback

Around half (53%, n=54) of the navigation tools have options for user feedback. These include contact information such an email address on the website for feedback (n=31), or a widget with a contact or feedback form (n=23).

### Glossary

Sixteen navigation tools include a glossary or similar, such as a list of terms with descriptions, explanations, or information about mental health conditions, AOD, or dementia (n=4). One navigation tool contains a list of acronyms or abbreviations but limited definitions, and one provides a glossary for rating IAR-DST domain impact. Three navigation tools provide a nonstigmatizing language guide (n=2) or a directory of language guides (n=1). Six navigation tools provide a glossary of mental health terms or conditions, and two provide a glossary of AOD with one including a drug information directory.

### Data Protection Policies

Australian privacy legislation mandates that websites display a privacy statement or privacy policy if they collect any information from consumers or visitors. Seventy-six percent (77/101) of navigation tools comply with this requirement by providing a privacy policy or statement on their website. Among the 24 navigation tools without such information available on the web, 10 are static navigation tools that do not collect any data, 11 provide privacy details through their developers’ websites (eg, PHNs), and the remaining three do not offer any privacy information on their websites.

## Discussion

### Principal Results

To our knowledge, this is the first study of Australian digital mental health navigation tools and their characteristics. The quantity and accessibility of mental health care navigation tools, or those encompassing mental health care services, has exponentially increased in recent years, from just a handful of tools in 2020 to more than 100 available in 2024. The large majority of these tools have been built with the consumer in mind. The research team was unable to locate or access any navigation tools specifically designed for planners.

Mental health care navigation tools are constantly and rapidly evolving with technological advances improving access to information and support. Despite this progress, there are persistent gaps and challenges that could be addressed to enhance their usefulness. These include varying quality in existing mental health care navigation tools, and a lack of standardization and definitions, leading to ambiguity about the type of care provided. This uncertainty affects individuals when choosing a service provider and health care professionals when referring consumers to service providers [22,23]. Tools ostensibly designed to facilitate access in fact could perpetuate further confusion.

### Comparison With Prior Work

Previous research indicates that individuals can find it challenging to access and use appropriate navigation tools, with the process being overwhelming and confusing [10]. To reduce this confusion, a system for navigating navigation tools appears to be necessary, akin to the Transcultural Mental Health Centre Directory of Directories in New South Wales, Australia.

The lack of accuracy or of tailored support in existing navigation tools remains a major challenge in accessing timely and appropriate mental health treatment and support [10]. Of the identified navigation tools, only 6 included a self-assessment screening tool capable of recommending services based on an individual’s mental health condition and required level of care. Improving the accuracy of navigation tools and information regarding types of service provision can significantly impact the effective linkage of individuals to suitable treatment and support services.

Locating information is a significant challenge in contemporary information systems [24]. The user interface of navigation tools can act as a barrier or facilitator to accessing information. Dieberger and Frank [24] argue that navigation is difficult unless the information space is designed in an understandable manner. Currently, Australian digital mental health directories are not easily navigable. Many of the navigation tools required significant searching and digital searching skills to locate required information, or to access a map of services and locations, and would benefit from a more user-friendly and intuitive design [13]. The three-click rule is an informal guideline in web design that pertains to the navigation of a website. According to this rule, users should be able to locate any desired information on a website within 3 mouse clicks [25]. The rationale is that users tend to experience frustration and often abandon a site and are unlikely to return if they are unable to find the information they are looking for within three clicks [25-27].

Only 30% (n=31) of navigation tools provided options such as language translation, or assistance with screen reading. Equitable access to mental health care service information is crucial for individuals who have low digital or health literacy, have a disability, or are from culturally and linguistically diverse backgrounds. Therefore, the design of the navigation tool and accessibility options such as language translation, large print, and screen reader functionality are paramount in addressing their needs [28,29].

The usefulness of existing navigation tools is further hindered by the limited availability of information about waiting times to access support and treatment from mental health care services [13]. Fewer than half of the navigation tools provided this information. Previous reviews have identified extensive waiting lists as a barrier for individuals seeking mental health support [30-33]. The estimated waiting time to access a service and the availability of services for new patients are crucial pieces of information for individuals seeking support and for health professionals referring individuals to services. The absence of information about available services, wait times, and client entry requirements is a major barrier to timely access to appropriate mental health services [30].

According to De Croon et al [34], health recommender systems represent a specialized application of recommender systems, using medical data, user input, and advanced algorithms to offer customized suggestions for improving health outcomes and wellness. One notable category of health recommender systems is the treatment recommender system, which furnishes specific recommendations for medical treatments, therapies, or interventions based on an individual’s health condition and medical history. Incorporating a health recommender system in navigation tools can help to address the challenges associated with varying health or digital literacy, information overload, and uncertainty about the quality of available information by providing personalized and tailored navigation tool recommendations. By leveraging these capabilities, health recommender systems hold the potential to significantly enhance personalized health care navigation, ensuring that recommendations align with each individual’s unique health profile and needs.

### Recommendations

#### Framework

Given these findings, it is essential to address the limitations of existing navigation tools to improve their quality and ensure greater standardization across platforms. Therefore, we have drawn on the NSQDMHS as a framework for developing recommendations to enhance these tools. Although the standards do not specifically address digital navigation tools, they provide a valuable foundation for establishing best practices and guidelines to ensure that such tools meet high standards of safety, quality, and effectiveness in mental health care navigation.

##### Care Planning: NSQDMHS Partnering With Service Users in Their Own Care 2.04

Integrating self-assessment screening tools and a health recommender system into navigation platforms to provide personalized service recommendations based on an individual’s mental health condition and required level of care will enhance the accuracy and tailored support provided by these tools. Fostering collaboration with mental health care professionals and organizations to validate the accuracy and effectiveness of the navigation tools would provide appropriate treatment and support services (eg, psychosocial services).

Ensuring that navigation tools offer comprehensive information on available models of care, including details on treatment modalities, specialties of mental health care providers, and specific support programs, will enable individuals to make informed decisions regarding their mental health care.

### Accessibility, Usability, and Digital Literacy

#### NSQDMHS Partnering With Service Users in Design and Governance 2.11—Accessibility

Information about accessible amenities and facilities must be available for each service. This should cover aspects such as wheelchair accessibility, availability of interpreters, and other accommodations relevant to diverse needs. Providing this comprehensive accessibility information empowers individuals to confidently select services that can effectively address their unique mental health requirements and preferences. Clear and inclusive information is essential in fostering a supportive environment, helping to ensure that individuals can access the mental health support they need without barriers.

Waiting time information must be provided for accessing mental health care services within the navigation tools. This should encompass estimated waiting times and the availability of services for new clients. Providing this information enables individuals seeking mental health care to make well-informed decisions about where to access services based on their specific needs and the anticipated wait times. Clear information about waiting periods helps ensure that individuals can select mental health services that align with their urgency and preferences, fostering a more responsive and supportive health care environment.

#### NSQDMHS Health and Digital Literacy 2.05 and 2.06—Communication That Supports Effective Partnerships

Interfaces for mental health services that are intuitive, user-friendly, and compatible with assistive technologies should be designed. The navigation tools must include multilingual support and language translation features to accommodate diverse linguistic needs. Options for adjustable text sizes and a clear, organized layout to enhance readability must be provided. Compatibility with screen readers and other assistive devices must be ensured. Additionally, the content and design must be developed with cultural sensitivity and inclusivity in mind, using plain language for instructions and user guides to make accessing mental health resources as straightforward and welcoming as possible. This approach will help ensure that all individuals, regardless of their abilities or backgrounds, can effectively navigate and use mental health services.

#### NSQDMHS Partnering With Service Users in Design and Governance 2.10—Usability

Information about mental health services must be accessible to individuals with varying levels of digital literacy. This can be achieved by providing clear, straightforward instructions and incorporating user-friendly features that assist those with limited digital skills. Navigation tools must be designed with intuitive interfaces and they must be accessible across various devices and platforms. Key features should include easy-to-use search functions, clearly categorized services, and accessible maps of service locations. Focusing on user-friendly design can enhance the usability of mental health resources, making it easier for individuals to find and access support.

Interactive features such as a chatbot or live chat option must be incorporated for mental health services, as these can be preferable for individuals with lived experience of mental health conditions who may find text-based interactions less confrontational than telephone calls. Additionally, conducting user testing with diverse groups—including those with various mental health experiences—can provide valuable feedback on the usability of the navigation tool. This feedback is crucial for making iterative design improvements and addressing specific usability issues, ultimately enhancing the overall user experience and making it easier for individuals to access mental health support.

### Quality and Accuracy

#### NSQDMHS Safety and Quality Systems 1.13 and 1.14—Feedback and Complaints Management

To enhance the quality and effectiveness of mental health navigation tools, protocols for consistently gathering feedback from service users regarding their experiences must be implemented. This input should be actively used to improve service delivery and outcomes. Additionally, a robust complaints management system must be established, clearly outlining the process, timeline, and steps for addressing feedback or complaints, ensuring transparency and accountability in responding to the concerns of individuals receiving mental health care.

#### NSQDMHS Safe Environment for the Delivery of Care 1.36—Continuity and Updates

A system for regular updates and maintenance of the navigation tools must be implemented to uphold the currency, accuracy, and reliability of the information provided. This is essential for users who depend on these tools to make informed decisions about accessing appropriate mental health care services, support, and resources.

Implementing these recommendations can significantly improve Australian digital mental health navigation tools, making them more user-friendly, accessible, equitable, and intuitive. By enhancing the accuracy and tailored support, these tools will better address the challenges users face in navigating mental health resources. Ultimately, this will strengthen the connection between individuals and timely, appropriate mental health care treatment and support services.

### Limitations

A systematic web search was conducted to identify navigation tools. Although the search methods identified a large number of navigation tools, there may be other navigation tools that were missed. Only Australian navigation tools that are available and accessible on the web were included in the infoveillance study. There are potentially paper-based or private digital navigation tools that are used by health care professionals or care navigators of which the research team did not have knowledge or to which they lacked access.

As stated, there are no standardized evaluation criteria for conducting reviews and assessment of the quality and accuracy of navigation tools, or the domains for different user groups. For example, the characteristics of a useful navigation tool for planners are different from the characteristics that are important for mental health care consumers. Therefore, a quality appraisal of navigation tools was not undertaken.

The risk of bias with an expert panel lies in the potential for subjective judgments or preferences of individual panel members to influence the group’s conclusions or recommendations. This bias can occur due to factors such as personal opinions, conflicts of interest, or professional affiliations that may sway the panel’s collective decision-making process. To mitigate this risk, composition of the panel was carefully considered to include diversity in perspectives or expertise, to reduce the risk of overlooking of alternative viewpoints or relevant evidence.

### Conclusion

Navigation tools have become seen as the solution to the complexity and fragmentation of Australia’s mental health system. Paradoxically, exponential growth in these tools risks perpetuating confusion over clarity. It is critical to address the limitations of existing tools to enhance quality and provide a level of standardization across all mental health system navigation tools. The current focus on mental health care reform in Australia is centered around the challenge of access to care for individuals with mental health concerns and strengthening services’ capacity to deliver appropriate, quality care across Australia [35]. Enhanced digital navigation tools possess a distinct capacity to influence this setting, with the aim of enhancing the accessibility of the health care system, minimizing care wastage, and promoting the well-being of individuals in need.

This study findings’ suggest a need for quality standards in digital mental health care navigation tools. These standards would enhance quality and accuracy and aim to eliminate ambiguity. Users would have increased confidence in selecting service providers. Additionally, it would aid health professionals in referring individuals to mental health care services.

The Australian Commission on Safety and Quality in Health Care and authors of the NSQDMHSs, which aim to improve the quality of digital mental health service provision, would be well placed to introduce these standards.

A potential next step includes compiling a national directory of mental health care navigation tools and their domains which will have the following benefits:

- Centralized resource: It creates a centralized resource for individuals seeking mental health care navigation tools, making it easier for them to find and access relevant tools.
- Comparative analysis: Having a directory allows for a comparative analysis of different tools and their features, enabling users to make informed decisions about which tool best suits their needs.
- Visibility and awareness: It increases visibility and awareness of available mental health care navigation tools among both users and health care professionals, potentially increasing their use.
- Quality assessment: It provides an opportunity to assess the quality and of various tools, helping to identify best practices and areas for improvement in digital mental health care navigation.
- Research and development: Researchers and developers can use the directory as a reference for understanding the landscape of existing tools and identifying gaps or areas for innovation in mental health care navigation technology.

## Abbreviations

AOD: alcohol and other drugs
GP: general practitioner
IAR-DST: Initial Assessment and Referral Decision Support Tool
NSQDMHS: National Safety and Quality Digital Mental Health Standards
PHN: Primary Health Network
